# Effects of Nuclear Motion on the Photoinduced Interfacial Charge Transfer Dynamics at a NiO/P1 Photocathode

**DOI:** 10.1021/acs.jpcc.4c08758

**Published:** 2025-03-27

**Authors:** Titus de Haas, Kaijian Zhu, Joannes M. van der Sterre, Yusen Luo, Guido Mul, Francesco Buda, Annemarie Huijser

**Affiliations:** 1 Leiden Institute of Chemistry, Leiden University, Einsteinweg 55, P.O. Box 9502, Leiden 2333 CC, The Netherlands; 2 Photocatalytic Synthesis group, MESA+ Institute for Nanotechnology, University of Twente, Enschede 7500 AE, The Netherlands; 3 School of Energy and Environment, City University of Hong Kong, 83 Tat Chee Avenue, Kowloon, Hong Kong SAR 999077, China

## Abstract

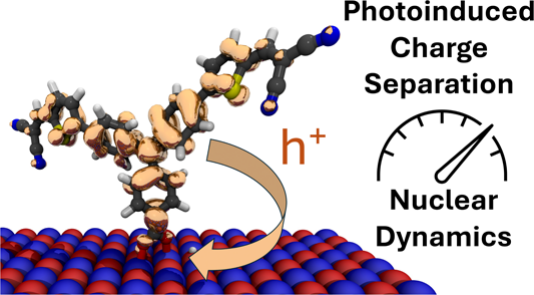

The performance of dye-sensitized photoelectrochemical cells is presently limited by the photocathode component. Here, we investigate the impact of nuclear dynamics on the photoinduced charge separation of the benchmark NiO/P1 system (P1 = 4-(bis-4-(5-(2,2-dicyano-vinyl)-thiophene-2-yl)-phenyl-amino)-benzoic acid). Transient absorption (TA) studies in aqueous environments with different viscosities show that photoinduced hole injection either proceeds ultrafast (<100 fs) or in a sub-ps time window. We assign the fastest component to a surface species strongly coupled to the NiO. Interestingly, the slower injection component and charge recombination are slowed down considerably in more viscous media. Quantum-classical dynamics simulations of a system with the dye standing perpendicular to the surface yield an injection lifetime remarkably close to the slow component from kinetic modeling of the TA results. Simulations including nuclear thermal motion yield a 2-fold increase in hole transfer rate compared to simulations on fixed nuclei, highlighting the role of nuclear motion and providing new design principles for dye-sensitized photocathodes.

## 1 Introduction

The development of efficient solar conversion devices is essential to advance the energy transition. Dye-sensitized photoelectrochemical cells (DSPEC) are attractive because of their tunable properties and capability to directly convert solar energy into H_2_ through water splitting or into other fuels via CO_2_ reduction.^1,2^ In a typical DSPEC cell, both photoanode and photocathode consist of a wide bandgap nanoporous semiconductor functionalized with light-absorbing dyes and catalysts. On the photocathode side, holes are injected from the dye into the p-type semiconductor, typically NiO, while electrons are transferred to the catalyst to drive the reaction. On the photoanode side, electrons are transferred into the n-type semiconductor, commonly TiO_2_, while holes migrate to the catalyst. However, due to the lower hole mobility of NiO compared to the higher electron conductivity of TiO_2_, charge recombination occurs much faster in the photocathode than in the photoanode.^3^ The photoelectrode with relatively low performance limits the overall efficiency of the device. Therefore, the efficiency of the photocathode is substantially lower than that of a photoanode,^4^ limiting the overall performance of a DSPEC and highlighting the need to develop more efficient photocathodes.

The choice for NiO as p-type semiconductor is motivated by its electronic energy levels and chemical stability.^5^ Despite numerous efforts focused on the NiO nanostructure and doping, and the design of new dyes and catalysts,^3^ improving the efficiency of NiO-based photocathodes remains a challenge. Hole injection from the photoexcited dye into the NiO typically occurs on a sub-ps time scale^6^ and should be followed by electron transfer from the dye into the catalyst.^7^ However, hole injection does not necessarily occur with close to unity quantum yield^8^ and also detrimental charge recombination between dye radical anions and holes injected into the NiO occurs fast, typically in a ps-ns time window.^9^ The photocathode performance is well-known to depend on the NiO nanostructure,^10^ doping,^7,9^ and the chemical structures of the dye and catalyst.^7,11−17^ Also the nature of the electrolyte and the composition of the electrochemical double layer play an important role in the performance and interfacial photodynamics.^18^

Theoretical studies by several groups on a variety of molecule-semiconductor photoanode systems have emphasized the key role of the nuclear dynamics in the interfacial photodynamics.^19−21^ These studies highlight that nuclear motion can facilitate nonadiabatic charge transfer channels which dominate the injection process when the electronic coupling between donor and acceptor moieties is low. Prezhdo et al. studied Ru complexes chemisorbed onto Ta_2_O_5_, either via COOH or PO_3_H_2_ anchoring groups. Nonadiabatic quantum-classical molecular dynamics (MD) simulations based on the Extended Hückel (EH) theory indicate that photoinduced electron transfer from Ta_2_O_5_ into the Ru complex is accelerated by COOH anchoring groups due to strong nonadiabatic coupling. The COOH anchoring groups promote charge transfer with higher frequency vibrational modes than the PO_3_H_2_ anchoring groups. Quantum decoherence appears to counteract this effect to a small extent, because of a faster decay in the COOH-anchored system compared to the PO_3_H_2_ tethering.^22^ Monti et al. studied the interfacial photoinduced electron transfer for a terrylene dye anchored on TiO_2_ by a phenyl-amide-phenyl molecular rectifier bridge. They found that the inclusion of nuclear dynamics is crucial to observe the process, and the electron propagation shows oscillatory features that correlate with interatomic distance fluctuations in the bridge.^23^ Similarly, Torres and co-workers have investigated a TiO_2_ surface sensitized with a perylene dye and observed that nuclear motion couples to the electron wave packet propagation.^19^ The role of nuclear-electronic coupling in intramolecular charge transfer has also been studied extensively for ruthenium-based dye compounds. These works have shown through both experimental and computational methods that specific molecular vibrations can couple with electron and hole dynamics in these systems.^24−27^

A wide variety of dyes have been investigated for the photosensitization of NiO for application in dye-sensitized solar cells, as reviewed by Gibson in 2019.^28^ Providing these complexes are stable in aqueous conditions, they can also be applied in NiO-based photocathodes. An important step forward was realized by the development of donor–acceptor (D-A) dyes, which consist of an electron donor (D) linked by a π-conjugated bridge to an electron acceptor (A). The complex is designed to spatially separate the electron and hole following electronic excitation and thus delay charge recombination, and is normally functionalized with one or more anchoring group(s) to enable chemisorption on metal oxides. As the photoinduced spatial separation of the electron and hole changes the dipole moment of the dye, the complex is typically stabilized by twisting of the donor relative to the acceptor.^29^

In the present work, we explore the photoinduced hole injection and charge recombination dynamics in a benchmark D–A dye chemisorbed on NiO. In particular, the role of nuclear motion on the photoinduced interfacial dynamics is investigated by combining EH-based quantum-classical dynamics simulations with femtosecond transient absorption studies in aqueous environments with different viscosities. The NiO is functionalized with the D–A dye P1 [4-(bis-4-(5-(2,2-dicyano-vinyl)-thiophene-2-yl)-phenyl-amino)-benzoic acid] by chemisorption via the COOH anchoring group. The P1 dye combines a triphenylamine donor unit linked via thiophene bridges to two dicyanovinylene acceptors. We observe that photoinduced hole injection either proceeds on an ultrafast time scale, during which the process is driven by electronic coupling, or in a sub-ps time window where nuclear motion is critical for the injection process. Especially the fluctuations in the dihedrals between the donor and acceptor moieties of the dye, and fluctuations in the carboxylate O – surface Ni distance appears to be important for photoinduced hole injection, although other nuclear dynamics likely play a role as well. Also, charge recombination depends on the structural dynamics and slows down in more viscous media. Our work highlights the importance of understanding the interplay between nuclear dynamics and photoinduced charge separation and recombination, which is essential for the development of photocathodes where structural flexibility is tailored for efficient solar to fuel conversion.

## 2 Methods

### 2.1 Sample Preparation and Transient Absorption Experiments

The sample preparation and femtosecond transient absorption setup has been described in detail earlier.^9^ The 800 nm pulse with a pulse duration of 35 ± 1 fs (full width at half-maximum) was generated using a Coherent Legend Ti:sapphire amplifier. The 500 nm pump beam was generated by sending the major part of the 800 nm beam into an optical parametric amplifier (Coherent, Opera). The time resolution is ca. 100–150 fs.^9^ Polyethylene glycol (PEG) was purchased from Sigma-Aldrich (BioUltra, 1000, *M*_n_ 950–1050) and dissolved into Milli-Q water with a concentration of 4.5 wt %.

### 2.2 Computational Methods

**Scheme 1:**
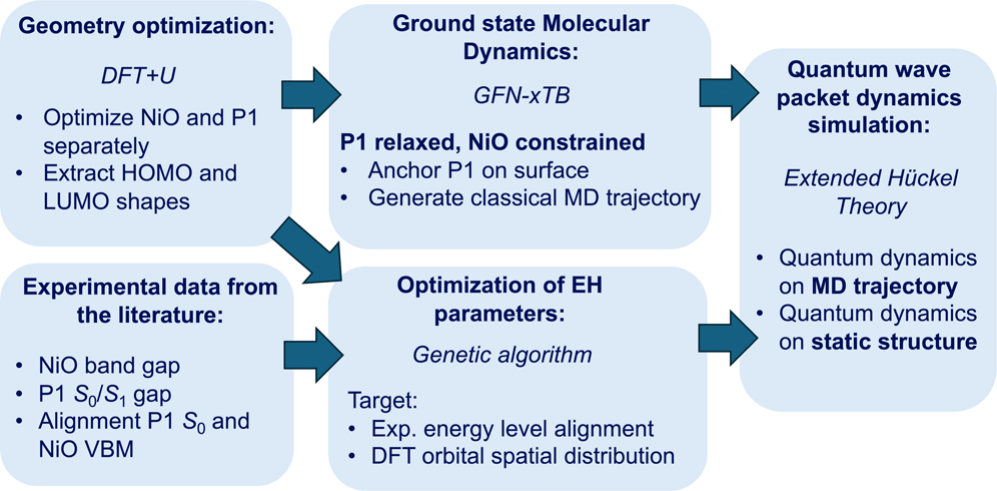
Schematic Overview of the Workflow Used for Photoinduced Hole Injection Simulations on the P1/NiO System VBM = valence band maximum, MD = molecular dynamics, EH = Extended Hückel.

Multiple computational methodologies were combined to study the electronic and structural factors that govern interfacial hole-transfer dynamics between the photoexcited dye P1* and the NiO surface. A schematic overview of the employed computational workflow is provided in Scheme 1. Density functional theory (DFT) was used to optimize both the geometries and electronic structure of P1 and the NiO semiconductor slab (details provided in the SI, sections SI.1, SI.2 and SI.7). The obtained dye and slab structures were used to build the configurations discussed in section 3.2. Nuclear classical dynamics simulations were performed of the dye molecule on the fixed DFT-optimized NiO using the self-consistent GFN-xTB tight-binding Hamiltonian.^30^ We note that these simulations did not include explicit solvent. The Coulomb integral parameters of the employed EH model were optimized to reproduce the experimental energy level alignment of the NiO/P1 interface and the spatial distribution of the P1 HOMO and LUMO in comparison with the corresponding DFT orbitals. This optimization procedure targeted the NiO band gap, the P1 HOMO–LUMO gap and the relative alignment of the P1 HOMO with the NiO valence band maximum (VBM), as discussed further in section 3.2.1. Details of the EH parameters optimization are provided in SI.8. Finally, the photoexcited electron and hole wave packet quantum dynamics were propagated either on a static nuclear geometry or on a precomputed nuclear trajectory using an EH approach. We have used a similar strategy based on semiempirical calculations in earlier work to describe photoinduced electron transfer dynamics in a dye-TiO_2_ interface.^21^ The details of the wave packet propagation schemes are explained in the papers by Rego and Batista.^31,32^ Details on the applied computational methodologies are provided in the SI, sections SI.1-SI.4.

## 3 Results

### 3.1 Femtosecond Transient Absorption Results

To investigate the effect of nuclear dynamics on photoinduced charge separation and recombination, femtosecond transient absorption (TA) studies have been carried out using 500 nm pump pulses, predominantly exciting the π-π* transition of the P1 dyes.^13^ We compared the photodynamics of NiO/P1 in H_2_O and NiO/P1 in H_2_O with 4.5 wt % polyethylene glycol (PEG), with the latter increasing the viscosity of the solvent and, therefore, likely slowing down the nuclear dynamics of the P1 molecule. Anchoring of the P1 dye onto the NiO surface via its COOH group causes deprotonation.^33,34^ The P1 molecules are solvated by H_2_O and PEG molecules, and H_2_O molecules are likely adsorbed onto the NiO surface.^18^ Dissociative H_2_O adsorption onto NiO has been reported to result in the formation of various Ni–OH surface species.^35^

**Figure 1:**
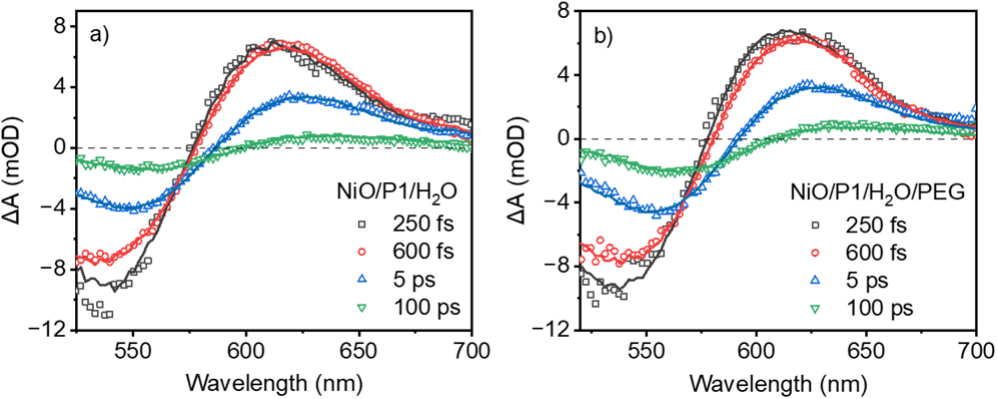
TA spectra for NiO/P1/H_2_O (a) and NiO/P1/H_2_O/PEG (b) at selected time delays after 500 nm excitation. The solid lines indicate fits from target analysis.

Figure 1 shows the TA spectra at various time delays for NiO/P1 in H_2_O (a) and in H_2_O/PEG (b) and fits from photophysical modeling (see below). All spectra show a negative signal below ca. 600 nm due to ground state bleach (GSB) of the photoexcited P1 dye (P1*). Also stimulated emission (SE) by P1* may contribute to the early time negative TA signal. Tian and co-workers observed SE at around 660 nm.^6^ Fluorescence up-conversion studies by Gustavsson et al. showed a signal at around 600 nm, decaying on a sub-ps time scale due to electronic relaxation from the bright Franck–Condon state into a low-emitting charge-transfer state.^36^ The positive TA signal around 610 nm at early times and red-shifting in time toward ca. 635 nm can be assigned to four distinct species. The first two are related to the dye: P1* has an absorption band centered around 550 nm and the P1 radical anion (P1^•-^) formed after hole injection into the NiO around 615 nm.^12,13^ The other two are due to redox processes in the NiO induced by photoinduced hole injection, resulting in Ni^3+^ and Ni^4+^ centers contributing to the absorption above 650 nm.^37^ Photoinduced hole injection by P1* resulting in P1^•-^ formation occurring beyond the TA time resolution (100–150 fs) contributes to the red-shift in TA spectrum with time observed here. The TA signals decay on a ps-ns time scale due to charge recombination.

**Figure 2:**
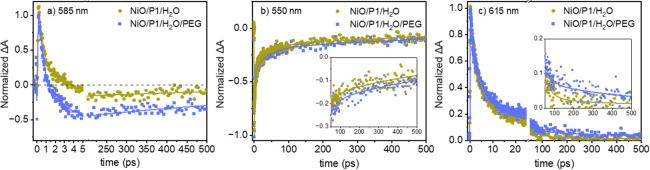
Normalized TA kinetic traces following 500 nm excitation for NiO/P1/H_2_O and NiO/P1/H_2_O/PEG at 585 (a), 550 (b), and 615 nm (c). The solid lines indicate fits from target analysis.

Figure 2a shows the early time normalized TA kinetic traces at 585 nm of NiO/P1 in H_2_O and H_2_O/PEG. The P1* signal at this wavelength persisting until a few ps exceeds that of the GSB&SE, resulting in a positive overall TA signal at early times. The signal decays faster in H_2_O/PEG, suggesting a shorter P1* lifetime caused by faster hole injection. Figure 2 also compares the kinetic traces at 550 nm (b) and 615 nm (c) of NiO/P1 in H_2_O and in H_2_O/PEG. The signal at 550 nm beyond a few ps is dominated by GSB, which is indicative of charge recombination and seems to decay slightly slower in H_2_O/PEG. This difference is clear at 615 nm, where the signal is predominantly due to P1^•-^. These observations are consistent with our photophysical modeling discussed below.

**Figure 3:**
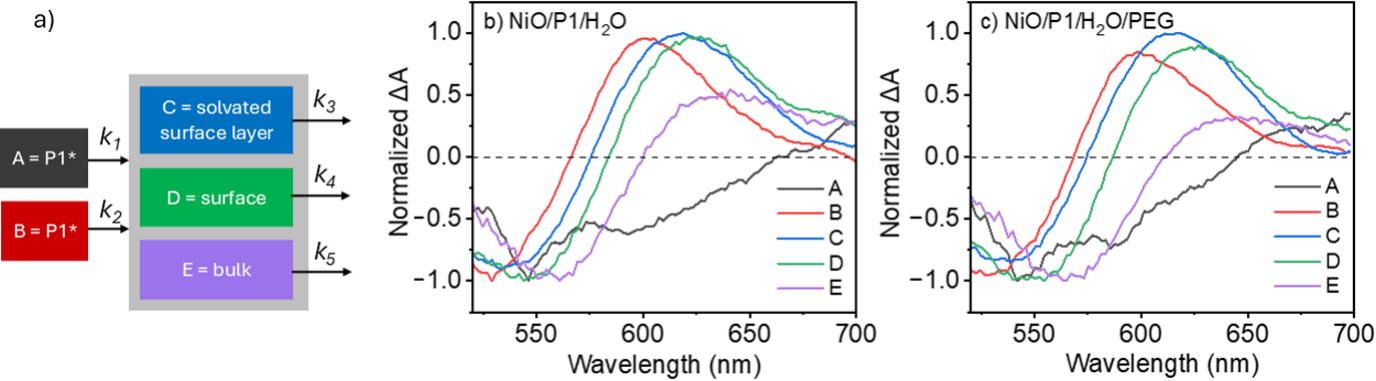
Photophysical model used for target analysis of the TA data (a) and obtained species associated spectra (SAS) for NiO/P1/H_2_O (b) and NiO/P1/H_2_O/PEG (c).

We performed target analysis using the program Glotaran.^38^Figure 3a presents a photophysical model based on five components that explains the TA data well. Photoinduced hole injection by P1* into the NiO either occurs ultrafast with rate constant *k*_*1*_, or proceeds on a sub-ps time scale with rate constant *k*_*2*_. Species A is a result of P1* GSB, SE and excited state absorption, with GSB and especially SE dominating the TA signal. In contrast, species B does not show such strong SE contribution, consistent with the work of Gustavsson et al. with the fluorescence of P1* not only being quenched due to hole injection into NiO, but also by intrinsic sub-ps electronic relaxation from the bright Franck–Condon state into a low-emitting charge-transfer state observed for P1 on insulating Al_2_O_3_.^36^ The time constants (Table 1, τ_*x*_ = 1/*k*_*x*_) qualitatively agree with the time constants obtained from quantum-classical dynamics simulations presented in the next section 3.2. The species associated spectra for each component are shown in Figures 3b and 3c. The τ_1_ is slightly shortened in the presence of PEG, consistent with the faster decay shown in Figure 2a. Although the difference is small, it might be a result of a more favorable initial P1 geometry for photoinduced hole injection induced by the PEG. In contrast to τ_1_, the value for τ_2_ is higher in H_2_O/PEG compared to H_2_O. We attribute this difference to the higher solvent viscosity in the presence of PEG, which likely slows down the nuclear dynamics of the P1 dye. Slow nuclear dynamics delay hole injection, as is demonstrated in the quantum-classical dynamics simulations presented in the section 3.2.

**Table 1:** Time Scales Obtained from Target Analysis

	NiO/P1 in H_2_O	NiO/P1 in H_2_O/PEG
τ_1_ (fs)	76 ± 3	60 ± 3
τ_2_ (fs)	479 ± 13	506 ± 12
τ_3_ (ps)	3.6 ± 0.04	3.9 ± 0.09
τ_4_ (ps)	33.3 ± 0.7	37.9 ± 0.7
τ_5_ (ps)	628.5 ± 15	725.5 ± 14

To properly describe charge carrier recombination, at least three components are needed to get a good fit. The fastest component (*k*_*3*_) is assigned to charge recombination between P1^•-^ and holes still present in the solvated NiO surface layer, the intermediate component (*k*_*4*_) to recombination with holes at the NiO surface and the slowest component (*k*_*5*_) to recombination with holes that have succeeded to escape into the NiO bulk. The spectra of the corresponding species C-E are included in Figures 3b and 3c and are clearly red-shifted relative to that of species B as a result of charge separation. Charge recombination is slightly slowed down in the presence of PEG, as also evident from Figure 2c, likely because of slower nuclear dynamics of the P1 dye due to the higher viscosity of H_2_O/PEG compared to H_2_O.

### 3.2 Quantum Chemical Modeling

**Figure 4:**
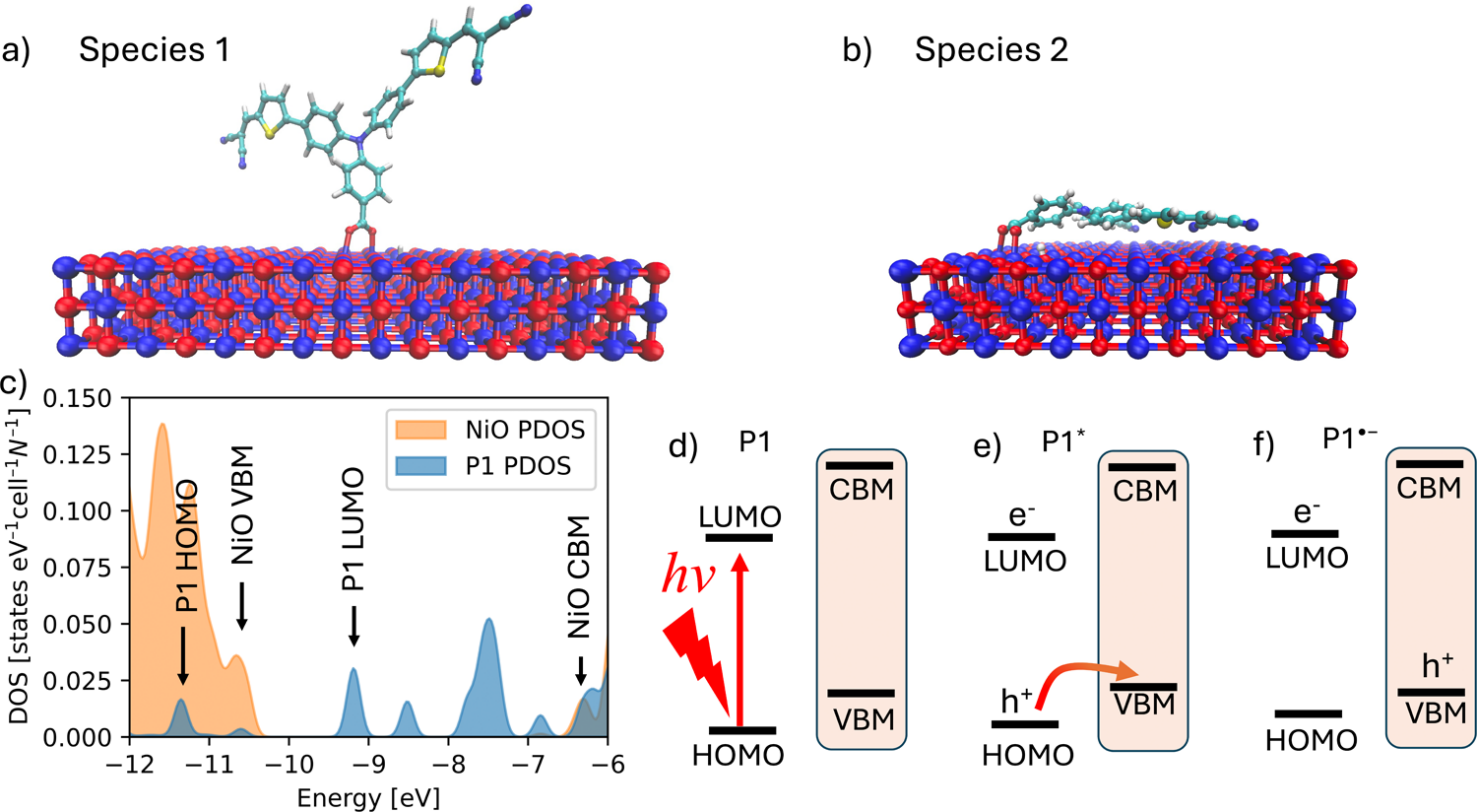
Visualizations of the P1 dye in the two configurations considered: (a) Species 1, standing perpendicular to the surface and (b) Species 2, lying flat on the surface. (c) Plot of the electronic density of states (DOS) of the optimized P1 dye on the NiO (100) slab, Species 1. The arrows indicate the positions of the P1 HOMO and LUMO orbitals and the NiO valence band maximum (VBM) and conduction band minimum (CBM) edges. The projected DOS (PDOS) of P1 and NiO were normalized by their respective number of atoms, N. (d–f) Schematic overview of the investigated P1 excitation and subsequent hole injection process.

Motivated by the two chemical species identified through kinetic modeling of the TA results, we considered two configurations of P1 in our quantum chemical modeling studies. In the first configuration (species 1), the dye is oriented perpendicular to the surface, while in the second (species 2), P1 lies flat with both tails physisorbed to the surface. Visual representations of these two P1 configurations are shown in Figures 4a and 4b. DFT-based geometry optimizations, performed on a small representative model system, indicate that species 2 represents a local minimum on the dye-NiO potential energy surface. In vacuum, this configuration is ∼ 30 kcal mol^–1^ lower in energy than the perpendicular configuration (see SI section SI.6, Table S2). The stability of the flat configuration arises from a strong π-stacking interaction between the aniline rings of the model dye and the NiO surface. However, this stabilizing interaction is expected to be significantly reduced in the solvated system due to the saturation of Ni atoms with physisorbed water molecules. We have also performed DFT-based MD on the same model with explicit solvent at room temperature, which show that the dye remains standing perpendicular to the surface on the ps time scale (see SI, section SI.6). Overall, we conclude that both species are likely to coexist on the surface. In both configurations, the carboxylate group is attached to the surface via a bidentate binding mode, with the COOH deprotonated and the excess proton bonded to an oxygen atom on the NiO surface. DFT studies of a similar dye with a carboxylate anchoring group have shown that this is the preferred binding mode on NiO (100).^33,34^

#### 3.2.1 Electronic Structure of the NiO/P1 Interface

The quantum wave packet dynamics is described by a semiempirical EH Hamiltonian.^39,40^ Prior to performing calculations, the parameters of this model were fine-tuned to reproduce the experimental energy level alignment on the interface structure.^41^ The HOMO orbital of the P1 has been estimated to be at ≈1.4 V vs NHE and the valence band maximum (VBM) for bulk NiO is found to be at ≈0.5 V vs NHE, providing a driving force of ≈0.9 V for hole injection by the photoexcited dye P1*.^42,43^ The 0–0 absorption of this dye has been estimated to be 2.24 eV, based on the intersection of absorption and emission spectra in acetonitrile.^44^ Bulk NiO has a band gap in the range of 3.4–4.3 eV, with the value depending on the measurement technique and the preparation method.^45−50^ These values have been employed to define optimization targets for the bulk NiO band gap and for the P1 HOMO and LUMO orbital energies. A genetic algorithm was used to fine-tune the Coulomb integral parameters of the EH model, producing the correct energetic alignment, while simultaneously maintaining the proper symmetry and spatial distribution of the P1 HOMO and LUMO.^23^ The details of the parametrization procedure are provided in section SI.8.

Figure 4c shows the electronic density of states (DOS) obtained from the EH calculation on the NiO/P1 interface for species 1 shown in Figure 4a. The DOS plot for species 2 can be found in the SI, Figure S6 and shows qualitatively the same energy level alignment. The model predicts a NiO band gap of ca. 3.9 eV, consistent with experimental data. Furthermore, it shows that the P1 HOMO is 0.9 eV below the NiO valence band maximum (VBM) and the P1 LUMO is 2.2 eV above the P1 HOMO. It should be kept in mind that despite the correct energy level alignment, the model does not reproduce the antiferromagnetic alignment of the Ni d-electrons and is, therefore, not able to reproduce the consequential anisotropy in the quantum wave packet dynamics in the semiconductor. However, this model should give a sufficient description of the electronic structure, as our primary focus is elucidating the effects of the P1 structural motions on the photoinduced hole injection rate rather than the diffusion of the wave packet inside the semiconductor itself. We note that despite neglecting the spin, the opening of the NiO band gap was naturally reproduced with the default parameters.^41^Figure 4(d-f) provide a schematic overview of the hole injection simulations discussed in this work. Initially, the ground state P1 dye is exposed to a light flash (Figure 4d), which excites the system to the first electronic excited state (Figure 4e). This excited state is subsequently quenched by hole transfer from P1* to NiO, forming the reduced dye radical anion P1^•–^ (Figure 4f).

#### 3.2.2 Hole Injection Simulations

After optimizing and equilibrating P1 in the configuration standing perpendicular to the surface (see Figure 4a, species 1), a 3 ps ground state MD simulation of the dye was performed at the GFN-xTB level. The obtained trajectory was then divided into three subtrajectories of equal length. Two EH based quantum dynamics simulations were performed on each subtrajectory: the first simulation incorporated nuclear motion from the MD, while the second simulation was performed using only the first MD snapshot as a static structure. By comparing these two simulations, the effect of thermal nuclear motion on the interfacial charge transfer dynamics was analyzed. Additionally, a single photoinduced hole injection simulation was performed on the optimized system where the dye lies flat on the surface (see Figure 4b, species 2). As the injection process for this species occurred on an ultrafast time scale, this simulation was carried out without including thermal nuclear motion.

**Figure 5:**
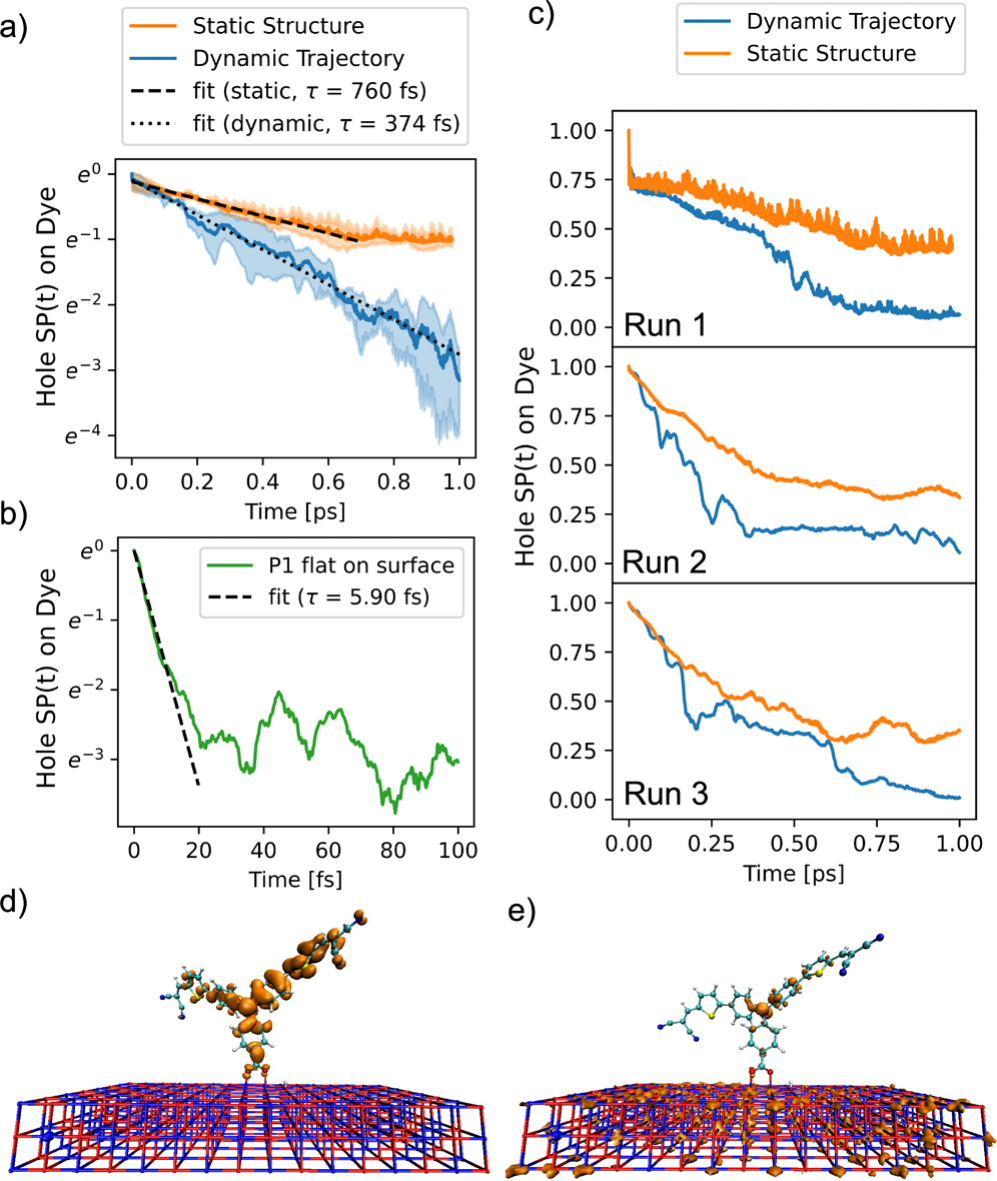
(a) Time-dependent survival probability (SP) of the hole density on the excited dye P1* averaged over static structures (red) or dynamic trajectories (blue) for species 1. The transparent areas represent one standard deviation centered around the mean value. Panel (b) shows the ultrafast injection in the case of species 2. Panel (c) provides the traces of the individual runs for species 1 on a linear *y*-axis scale. Panels (d) and (e) present visualizations of the hole wave packet density at 0 and 900 fs extracted from run 2.

Figure 5a presents the averaged hole survival probability (SP) for the species 1 photoexcited dye P1* over time, computed on the dynamic trajectories (blue) and on static structures (orange). Figure 5b presents the hole SP for the dye lying flat on the surface, while Figure 5c provides the injection traces for the individual trajectories of species 1, without averaging. The electron SP traces are provided in the SI, Figure S4. Figures 5d and 5e display the hole density in Run 2 before and after injection. It is clearly observed that the hole is localized in the P1 HOMO orbital prior to injection, while it is delocalized over the entire NiO surface after injection.

The averaged decay traces of the hole SP were modeled with an exponential decay function . The *m* parameter was introduced to account for the initial ultrafast injection on the first ca. 5 fs observed in Figure 5c, Run1. For P1 standing on the surface, this yielded lifetimes of 760 fs for the injection process on the fixed nuclei and 374 fs for the injection on the moving nuclei. This lifetime is remarkably close to the lifetime obtained for the second component by target analysis of the TA results (see Table 1). The fit for the injection on fixed nuclei was performed only on the first 690 fs, as the curve does not follow exponential behavior over the final 310 fs. Figures 5a and 5c highlight the critical role of nuclear dynamics on photoinduced hole injection in this system. Although all simulations predict a rapid initial injection process, the hole transfer rate on the static structures is rapidly diminished after approximately 66% of the charge density has been injected, resulting in an average SP on the dye of 34% after 1 ps. In contrast, the hole injection along the dynamic trajectories continues without stagnation during the 1 ps simulation, resulting in a 4% hole SP on the dye.

The first injection simulation with P1 standing on the surface (Run 1, Figure 5c) displays a remarkably fast initial injection, causing a 27% reduction of hole SP on the P1 dye within the first 5 fs. This ultrafast component is attributed to very rapid hole transfer to an electronic orbital which is partly delocalized on the NiO (see SI, section SI.10, Figure S5). It was found that for the initial structure of Run 1, and other nuclear geometries, this orbital energy becomes nearly degenerate with the P1 HOMO energy (*E*_HOMO_ – *E*_HOMO – 1_≈ 0.05 eV, see SI, Figure S5). The strong electronic coupling between these quasi-degenerate orbitals leads to an ultrafast charge transfer channel from the P1 HOMO to the NiO valence band.

Figure 5b presents the photoinduced hole-injection trace for the dye lying flat on the NiO surface. The charge transfer process for this species is observed to occur on a significantly faster time scale, with a sub-10 fs lifetime, which is nearly 2 orders of magnitude shorter than that of the P1 oriented perpendicular to the surface. This much faster injection process occurs because the hole density can diffuse directly from the P1 aniline core into the NiO surface, without evolving through the carboxylate anchoring group.

We now further investigate the role of thermal nuclear motion on the interfacial charge transfer dynamics, specifically for species 1, where the dye stands perpendicular to the surface. To analyze this effect, we look at the hole SP trace in the frequency domain. By Fourier transforming the velocity autocorrelation function of the SP time evolution, power spectra were obtained for the hole SP on fixed and moving nuclei. Similarly, a vibrational density of states (VDOS) was obtained from the nuclear trajectory.

**Figure 6:**
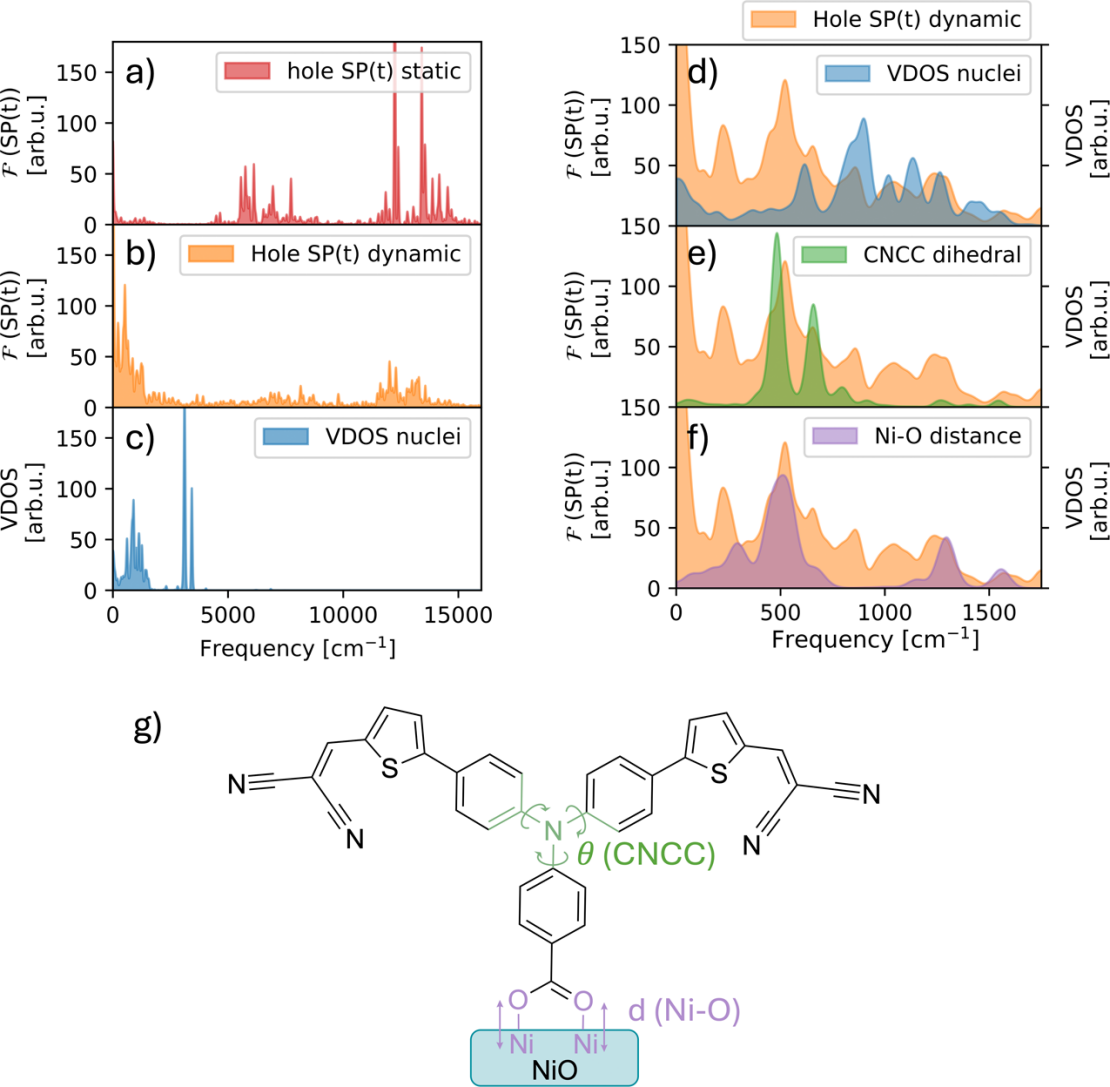
Frequency spectra associated with the hole transfer on fixed (a) and moving (b) nuclei, and the vibrational density of states (VDOS) of the nuclear trajectory (c). A frequency spectrum was computed for each run individually, and subsequently averaged to obtain the displayed figure. Panel (d) provides an overlay of hole SP frequency spectrum on moving nuclei and the VDOS of the entire trajectory. Additional overlays are provided for the same hole injection spectrum, but now compared with the VDOS computed by considering only the CNCC dihedral angle torsions (e) or the Ni–O distance (f) fluctuations. The relevant Ni–O distances and CNCC dihedral angles are shown in panel (g). Spectra for both Ni–O fluctuations and all three CNCC dihedral torsions (displayed in (g)) were averaged to produce the spectra displayed in (e) and (f).

Figure 6a shows the hole injection frequency spectrum for the quantum dynamics performed on the static structures of species 1. The spectrum is characterized by sharp peaks around 6000 cm^–1^, 12000 cm^–1^ and 13500 cm^–1^. These frequencies are attributed to fast Rabi oscillations of the hole wave packet between different electronic eigenstates mostly located on the P1 dye, but with a slightly different delocalization on the NiO slab. Such rapid Rabi oscillations were also observed in the simulations by Torres et al.^19^ Interestingly, the frequency spectrum of the injection trace on moving nuclei (Figure 6b) shows a much stronger signal in the 0–1700 cm^–1^ region. As evidenced by the vibrational density of states (Figure 6c), this is precisely the frequency range where nuclear vibrations are expected to play a role. Evidently, the propagation of the hole wave packet exhibits strong coupling to the nuclear motion in this range. In Figure 6d, the VDOS and hole SP frequency spectra are overlaid in the 0–1700 cm^–1^ region. The frequency spectrum of the hole injection peaks around 500 cm^–1^ and also contains several other characteristic frequencies. This observation indicates that a broad range of vibrations couple to the wave packet dynamics. To further investigate which vibrational modes are important, we extracted from the simulations the characteristic frequencies of specific geometrical features and found two modes that appear to be in resonance with the hole dynamics. Particularly, fluctuations of the CNCC dihedral angles (displayed in green in Figure 6g) and of the anchoring Ni–O distances (displayed in purple in Figure 6g) show high correlations with the injection process as evidenced by the spectra shown in Figure 6e and Figure 6f.

The influence of the CNCC dihedral angle on hole injection was further examined by visualizing the hole wave packet dynamics in the excited P1*. A video (supplied in the associated content), shows that the wave packet oscillates several times between the two tails of the P1* before eventually diffusing into the NiO surface. This behavior suggests that the specific geometric conformation of the dihedral angles at the P1 aniline core plays a critical role in directing the wave packet. Depending on the alignment of these dihedrals, the wave packet may either evolve toward the surface or remain confined to the opposite tail. Recent studies on ruthenium bipyridine dye complexes show that, upon photoexcitation, electron transfer dynamics between the two bipyridine ligands can occur on a similar sub-100 fs time scale as observed here.^25^

## 4 Discussion

We have performed quantum-classical dynamics simulations and femtosecond TA spectroscopy experiments to investigate the photoinduced hole injection dynamics at the NiO/P1 interface, which serves as a benchmark system for photocathodes. After excitation of the P1 π-π* transition using 500 nm pump pulses, the excited dye P1* is quenched by rapid (subps) hole injection into the valence band of NiO. Target analysis of the femtosecond TA data indicates that the photoinduced hole injection process in water is best described by a biexponential decay with lifetimes τ_1_ and τ_2_ of 76 ± 3 fs and 479 ± 13 fs, respectively. A good model for the subsequent charge carrier recombination process was obtained using three components with lifetimes of a few to several hundred picoseconds. The addition of PEG increases the values of τ_2_*-*τ_5_, which could be attributed to slower nuclear dynamics of the P1 dye.

Motivated by the observation of two characteristic lifetimes for hole injection in the TA results, two systems were considered in our quantum chemical modeling studies. While in the first system, the P1 dye stands perpendicular to the surface (see Figure 4a), the second system considers the dye as it lies flat on the surface (see Figure 4b). DFT calculations on a smaller model system confirm that the dye is flexible and could form strong π-stacking interactions with the NiO surface. Although this suggests that the P1 dye could adopt configurations in which the tails come near the surface, the real system probably does not collapse to the surface entirely due to the solvating effect of surrounding water molecules and due to the entropic gain associated with dissociation of the P1 tail from the surface. We attribute the fast component observed in the TA target analysis to a surface species in which the P1 aniline core is strongly coupled to the NiO surface layer. Quantum-classical dynamics simulations show that a P1 molecule that lies flat on the NiO surface exhibits a hole injection process characterized by a sub-10 fs lifetime. For the system with the dye standing on the surface, we observed that in specific geometric conformations, the electronic state corresponding to the hole occupying the P1 HOMO, becomes nearly degenerate with a state where the hole is delocalized on the P1 carboxylate bridge and the NiO surface. It was found that this quasi-degeneracy also leads to strong coupling between the P1* and the surface, providing a channel for ultrafast injection of roughly 27% of the hole density in ∼ 5 fs.

Exponential fitting of the photoinduced hole injection trace for the P1 dye standing perpendicular to the surface yielded a characteristic lifetime of 374 fs, in near-quantitative agreement with the slower component of the injection in H_2_O media. The 22% faster decay in the simulation compared to the TA results is within the error margin of the applied computational method and may be explained by several contributing factors. In a previous study, we demonstrated that including explicit solvent molecules can affect the nuclear conformations visited by the dye during the MD simulations, thereby influencing the injection rate.^21^ The absence of explicit solvation in the simulations increases the P1 flexibility, thereby enhancing the nonadiabatic coupling between donor and acceptor states, accelerating the decay process. Recent studies on photoinduced intramolecular charge transfer dynamics in a ruthenium-based chromophore have shown that explicitly accounting for solvent molecules can considerably slow electron and hole mobility due to solute–solvent polarization effects.^26^ Neglecting such polarization effects in our simulations could therefore result in an overly fast injection process. Finally, our model system shows a fast and isotropic diffusion of the hole wave packet into the NiO slab, whereas the actual injection process is constrained by the antiferromagnetically coupled spin layers in the NiO (111) plane. Once again, these approximations could contribute to the slightly faster injection in the model system compared to the TA measurements.

Both the TA measurements and the quantum-classical dynamics simulations highlight the importance of nuclear motion for the injection process. Our results support the idea that the increase in solvent viscosity is the underlying reason for the higher τ_2_-τ_5_ in H_2_O + PEG compared to H_2_O. We note here that the very small change in solvent reorganization energy due to a change in solvent polarity in the presence of PEG might also decrease the electron transfer rate according to Marcus theory. However, this slight change in polarity is not expected to play a role on the sub-ps time scales at which the solvent has not yet adjusted to the electronic excitation and the system is still out of equilibrium.

The performed quantum-classical dynamics simulations of the dye standing perpendicular to the surface reveal that the removal of nuclear motion doubles the injection time scale (τ = 374 fs on a moving trajectory *vs* τ = 760 fs on static structure) and leads to a stagnation of the hole transfer after ca. 69% injection. Fourier analysis of the hole transfer simulations on moving trajectory reveals that the spectrum of the injection trace is characterized by frequencies in the 0–1700 cm^–1^ range. In contrast, the injection is characterized by much higher frequencies, around 6000 cm^–1^ and above 12000 cm^–1^, when keeping the nuclei fixed. It thus appears that the hole dynamics are affected considerably by the nuclear motion of the dye. Specifically, torsional motions around the dihedral angles between the three aniline components in the P1 dye (see Figure 6g, green dihedrals), appear to be resonant with the hole density propagation. Prior to injection, the hole wave packet oscillates multiple times between the two P1 tails. We propose that a specific geometric conformation involving the dihedrals between the core aniline units directs the hole wave packet either toward the opposite P1 tail, or toward the NiO surface. In addition to these dihedral angle torsions, also fluctuations in the Ni–O bond lengths at the NiO/P1 interface appear to correlate with the photoinduced injection trace. Overall, the performed calculations underscore how the injection process is strongly coupled to nuclear motion of the dye. These findings align with the literature, where coupled electron–nuclear dynamics is a feature observed for charge transfer in molecules, materials and biological systems.^21,24−27,51−54^

## 5 Conclusions

In summary, we have performed quantum-classical dynamics simulations in combination with TA spectroscopy to investigate the effects of nuclear motion on the photoinduced hole injection dynamics at the prototype NiO/P1 photocathode interface. The injection process of the excited dye P1* into the NiO surface is best fitted with a bi-phasic exponential consisting of an ultrafast component (τ_1_ of 76 ± 3 fs, within the TA instrumental response) and a slower component (τ_2_ of 479 ± 13 fs). Based on quantum chemical modeling, we assign the fast component to P1 molecules that are strongly coupled to NiO, possibly through π-stacking interactions of the P1 tails to the surface. The slow component is assigned to P1 molecules standing perpendicular to the surface. Notably, the lifetime of this slow component depends on the solvent viscosity, as was shown by comparing the TA data of the NiO/P1 system in water and in water with 4.5 wt % PEG. Also charge recombination is slightly slowed down in more viscous media.

Quantum-classical dynamics simulations show excellent agreement with the TA results and further highlight the critical role of nuclear motion. Simulations of quantum hole dynamics on moving nuclei predict an approximately 2-fold faster injection rate compared to those on fixed nuclei. Subsequent frequency domain analysis shows that the fluctuations of the hole survival probability on the P1* are in resonance with the vibrational fingerprint of the dye molecule. Specifically, torsional degrees of freedom around the P1 tails appear to be correlated with the injection trace. These results provide detailed insights into the fundamental photochemistry at the NiO/P1 interface, offering valuable guidelines for the design of highly efficient dye-sensitized photocathodes.

## Supporting Information Available

Computational details on DFT-based NiO slab structure optimizations; optimization of the Hubbard-U parameter; DFT-based molecular dynamics of model dye-NiO system in explicit solvation; DFT optimization and TDDFT excitations spectrum of the P1 dye; optimization of the extended-Hückel parameters; electron dynamics simulations on static structures and dynamics nuclear trajectories; ordering of P1 HOMO and HOMO–1 states for different conformations sampled from MD (PDF)
(Video S1) Visualization of the hole injection process on the moving trajectory (Run 2) (MPG)

## Supplementary Material

jp4c08758_si_001.pdf

jp4c08758_si_002.mpg
