# Supplementary material for: Effects of Nuclear Motion on the Photoinduced Interfacial Charge Transfer Dynamics at a NiO/P1 Photocathode
Source: J Phys Chem C Nanomater Interfaces. 2025 Mar 27;129(14):6817–26. doi: 10.1021/acs.jpcc.4c08758 (PMC11998066; doi:10.1021/acs.jpcc.4c08758)
Supplement: Supplementary file 1 — jp4c08758_si_001.pdf [file jp4c08758_si_001.pdf]

SUPPORTING INFORMATION

Effects of Nuclear Motion on the Photoinduced Interfacial Charge Transfer Dynamics at a NiO/P1 Photocathode

Titus de Haas[1†], Kaijian Zhu[2,3†], Joannes M. van der Sterre[1], Yusen Luo[2], Guido Mul[2], Francesco Buda[1]* and Annemarie Huijser[2]*

[1]*Leiden Institute of Chemistry, Leiden University, Einsteinweg 55, PO Box 9502, 2333CC Leiden, The Netherlands*

[2]*Photocatalytic Synthesis group, MESA+ Institute for Nanotechnology, University of Twente, 7500 AE Enschede, The Netherlands*

[3]*School of Energy and Environment, City University of Hong Kong, 83 Tat Chee Avenue, Kowloon, Hong Kong SAR, 999077，China*

†*Contributed equally.*

**Contributed equally.*

*Corresponding Authors:

Francesco Buda, email: f.buda@lic.leidenuniv.nl, ORCID : 0000-0002-7157-7654

Annemarie Huijser, email: j.m.huijser@utwente.nl, ORCID: 0000-0003-0381-6155

Table of Contents

SI.1 Computational details on DFT-based NiO slab structure optimizations.* *S2

SI.2 NiO structure optimization*...*S3

SI.3 Ground state P1/NiO molecular dynamics simulations.* *S4

SI.4 Electron and hole wavepacket simulations.* *S4

SI.5 Optimization of the Hubbard-U parameter.* *S5

SI.6 DFT-based molecular dynamics of model dye-NiO system in explicit solvation*..........*S6

SI.7 DFT optimization and TDDFT excitations spectrum of the P1 dye.* *S8

SI.8 Optimization of the Extended-Hückel parameters*..*S9

SI.9 Electron dynamics simulations on static structures and dynamics nuclear trajectories.* *S12

SI.10 Ordering of HOMO and HOMO-1 states for different conformations sampled from MD.* *S13

SI.11 Extended Hückel-based DOS plot for P1 lying flat on surface.* *S14

SI.12 Appendix: The optimized EH parameters.* *S15

References* *S16

SI.1 Computational details on DFT-based NiO slab structure optimizations.

All optimizations were carried out using the Quickstep module in the CP2K8.2 software package.[1] The calculations applied the generalized gradient approximation (GGA) introduced by Perdew, Burke and Ernzerhof for the treatment of the exchange correlation potential with Grimme's third generation of dispersion corrections including a 22 Å cutoff and grid-smoothening.[2–5] A Mullikan based Hubbard-U parameter of 3.4 eV was applied on the Ni d-electrons to improve the description of the electronic structure over the standard GGA based DFT. More details on the optimization of the Hubbard-U parameter are provided in SI.5. The calculations employed a hybrid Gaussian Plane Wave (GPW) scheme as implemented in the Quickstep routine in CP2K8.2.[6] The Ni and O valence electrons were expanded in the DZVP-MOLOPT-SR-GTH basis, while the core-valence electron interactions were modeled with pseudopotentials developed by Goedecker, Teter and Hutter.[7–9] A cut-off energy of 400 Ry was used for the auxiliary plane wave basis. All calculations considered only the Γ-point. Optimizations of the electronic structure were converged to

$5 \cdot 10^{-7}$ H $cycle^{-1}$ and geometric structure optimizations were converged to root mean square values of 10^{-4} $Bohr^{-1}$ in the step size and 10^{-5} H $Bohr^{-1}$ in the forces.

SI.2 NiO structure optimization.

The bulk structure of NiO was optimized in a rhombohedral cell (α=β=γ=60°) containing 64 nickel and 64 oxygen atoms at the DFT+U level of theory in CP2K8.2.[1] Cell-optimizations yielded a quasi-perfect rock salt structure with an average cubic lattice parameter of 4.17 Å, which matches lattice parameters reported in literature.[10–12] Visualization of the spin-density demonstrates that the optimized electronic structure exhibits anti-ferromagnetic spin alignments in the (111) plane (see Figure S1). The bulk optimized rhombohedral was transformed to an orthorhombic box and cut along the (100) direction. The x and y dimensions of the box where preserved, but the z-axis was elongated to 40.0 Å, introducing a vacuum of 28.2 Å above the surface. Subsequently, a structure optimization was carried out in which the lowest layer of nickel and oxide atoms were kept at fixed positions to simulate the constraining effect of the bulk material. In line with previous studies, the (100) surface undergoes only minor rearrangements.[13–16] As a sanity check, slab optimizations were carried out also for the (111) surface. Also this optimization yielded a structure that aligns with reports in literature.[17]

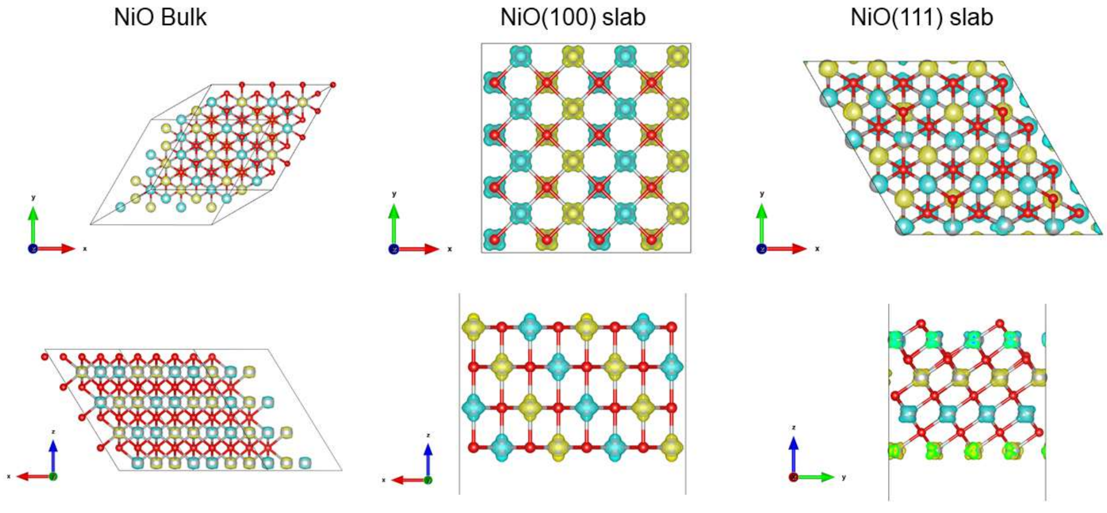

***Figure S1**: Visualization of the DFT+U optimized bulk and slab NiO structures including the spin density, demonstrating the anti-ferromagnetic spin alignment in the (111) plane for both phases.*

SI.3 Ground state P1/NiO molecular dynamics simulations.

The optimized NiO (100) surface slab was used to generate a 3-layer orthorhombic slab of 8 by 4 NiO units (192 Ni and 192 O atoms in total). In the center of the unit cell, the optimized P1 dye was attached to the surface through a bidentate binding mode where both carboxylate oxygens were attached to two distinct nickel atoms (see Figures S2c and S2d for a visualization of the system). Previous DFT studies of a similar dye with a carboxylate anchoring group have shown that this is the preferred binding mode on NiO (100).[16] Optimizations and molecular dynamics (MD) simulations of the dye attached to the surface were subsequently performed at the GFN-xTB level of theory, including Grimme's third generation of pairwise additive dispersion corrections.[4] The P1 structure was optimized on the surface, and initial conditions for a production run were obtained by performing a 5 ps equilibration run at 300 K. The timestep for this equilibration run was set to 0.5 fs and a constant temperature was maintained with the Canonical Sampling through Velocity Rescaling (CSVR) thermostat.[18] The timestep for the production runs was set to 0.1 fs. During the optimization, equilibration and production runs, the positions of the nickel and oxygen atoms of the NiO slab were kept fixed. We note that we have also attempted MD simulations with fully relaxed Ni and O atoms. However, we found that NiO rock salt structure was unstable during these simulations, resulting in nickel and oxygen dissociation from the surface. These unstable dynamics are likely a result of the limitations of GFN-xTB in properly describing the anti-ferromagnetically coupled spin layers in the NiO (111) plane. To validate the decision to neglect structural relaxations of NiO in response to the P1 dye anchoring, DFT-based MD simulations were performed on a smaller but representative model system. This system consisted of a 4-(diphenylamino)benzoic acid molecule on a NiO (100) slab of four layers of 8x8 NiO units and explicit water solvation. The simulation showed that neither the solvent nor the dye molecule had significant effects on the NiO surface structure, justifying the approach described above. These DFT-based simulations on the smaller model system are further discussed in SI.6.

SI.4 Electron and hole wavepacket simulations.

After the ground states nuclear trajectories were obtained, the evolution of the excited state electron and hole wave packets were simulated on the basis of the Extended Hückel formalism, as implemented in the DynEMol software.[19] For all simulations, the photoexcited

state was prepared by initiating an electron wave packet in the P1 LUMO and a hole wave packet in the P1 HOMO, which is justifiable considering that the 0-0 excitation in the P1 dye is largely dominated by the HOMO-LUMO transition (see SI.7). The time-step for the wave packet propagation was set to 0.1 fs, which is short enough to guarantee proper convergence of the electronic sub-system.[20] The quantum propagation of the wave packet is followed by calculating the time-dependent survival probability (SP) on the dye and on the NiO slab. This property is calculated by projecting the wave function at time t, on the atomic orbital basis of the molecular sub-system of interest.[21]

SI.5 Optimization of the Hubbard-U parameter.

As mentioned in the section SI.1.2, the calculations reported in the paper employed a Mulliken-based Hubbard U parameter of 3.4 eV to the Ni d-electrons to improve the description of the NiO electronic structure over standard GGA-DFT. As the goal of the DFT optimizations was only to obtain a structure for the subsequent quantum wave packet simulations, we focused on obtaining a correct geometry rather than reproducing an accurate band gap. With these considerations in mind, a series of 6 bulk phase cell-optimizations were performed, applying a Hubbard parameter of 1, 2, 3, 4, 5 and 6 eV, respectively. These cell-optimizations were performed on a rhombohedral box containing 64 nickel and 64 oxygen atoms. This series was later extended with additional optimizations applying a U parameter of 3.4 and 3.6 eV. Subsequently, a series of electronic structure optimizations was performed on a perfect NiO bulk structure with a lattice parameter of 4.17 Å, to extract the predicted band gap. The obtained lattice parameter and band gap energies are provided in Table S1. Based on these benchmark calculations, it was decided to perform the slab optimizations with a Hubbard U parameter of 3.4 eV, as this yielded a good compromise between the predicted lattice parameter and band gap properties.

Table S1. Lattice parameters and band gap energies for the performed NiO bulk phase optimizations with different Hubbard U values. Experimental data are shown in the last column.

Hubbard U parameter	1 eV	2eV	3 eV	3.4 eV	3.6 eV	4 eV	5 eV	6eV	*Exp.*
Lattice parameter (Å)	4.123	4.187	4.173	4.167	4.153	4.180	4.150	4.100	4.17[10–12]
Band Gap (eV)	1.48	1.64	1.93	2.10	2.19	2.39	2.90	2.75	3.4-4.0

SI.6 DFT-based molecular dynamics of model dye-NiO system in explicit solvation.

To investigate the structural response of the NiO surface to the anchoring of P1, DFT-based molecular dynamics were performed using a smaller model system. In this model system, P1 was represented by a 4-(diphenylamino)benzoic acid (4DPABA) molecule. 4DPABA represents a good model for the P1 dye as it contains the same benzoic acid anchoring group and triphenyl amine core. The same computational settings were employed as described in section SI.1 The optimized NiO (100) surface was used to generate a slab of 4 layers of 4 by 4 NiO units (total of 128 Ni and 128 O atoms). The 4DPABA molecule was considered to attach to the surface in a bidentate binding mode, as earlier studies from Pavone *et al* have shown that this is energetically more stable than the monodentate binding.[16] The carboxylate oxygen atoms were considered to bind to two distinct Ni atoms at the surface, while the dissociating carboxylate proton was considered to bind to the adjacent oxygen atom. Since each Ni atom hosts two unpaired electrons, two distinct binding modes are possible: one in which the two carboxylate oxygen atoms bind to two Ni atoms with the same spin polarization (αα-binding), and another where they bind to two Ni atoms with opposite spin polarization (αβ-binding). Furthermore, we considered two different surface species. The first species stands perpendicular to the NiO surface and is displayed in Figure S2a, while the second species lie flat on the surface and is displayed in Figure S2b. All interface structures were optimized in a cubic box with dimensions 16.6 by 16.6 by 24 Å. The energies associated with each species are reported in Table S2. Interestingly, the species were the 4DPABA dye lies flat on the surface was found to be ~30 kcal mol^{-1} lower in energy than the species where the dye stands perpendicular to the surface. The difference between binding two to nickel atoms of same or opposite spin appears to have an effect in the order of 1 kcal mol^{-1}.

Table S2. Relative energies for the four considered 4DPABA surface species.

	Energy (kcal mol^{-1})
standing perpendicular (αα)	30.4
standing perpendicular (αβ)	29.3
flat on surface (αα)	0
flat on surface (αβ)	0.9

After geometry optimizations of the 4DPABA-NiO interface, 129 water molecules were added to the simulation box with the PACKMOL builder tool as implemented in AMS2021.[22,23] The system was equilibrated for 1 ps, and subsequently propagated for another 3 ps at the DFT-MD level. During the dynamics, the CSVR thermostat was applied with a timestep of 50 fs.[18] Figure S2c-d presents a snapshot from the MD simulations. During the dynamics, the 4DPABA dye was found to remain strictly perpendicular to the surface and the bidentate binding mode was found to remain stable for the entire duration of the simulation. This indicates that the interconversion between the conformations - with the dye standing perpendicular or parallel to the surface - is associated with a barrier substantially higher than $k_B T$. Water molecules appeared to be strongly physisorbed to the surface, with the oxygen atoms consistently oriented towards the surface nickel atoms, while the hydrogens atoms formed strong hydrogen bonds with the surface oxygen atoms. In the course of the simulation, two water molecules underwent O-H bond dissociation, resulting in their chemisorption on the surface. The binding of the 4DPABA molecule appeared not to affect strongly the structure or the dynamics of the Ni and O atoms at the dye-surface interface. This result justifies the assumption of neglecting the structural motion within the NiO slab in the GFN-xTB simulations reported in the main manuscript.

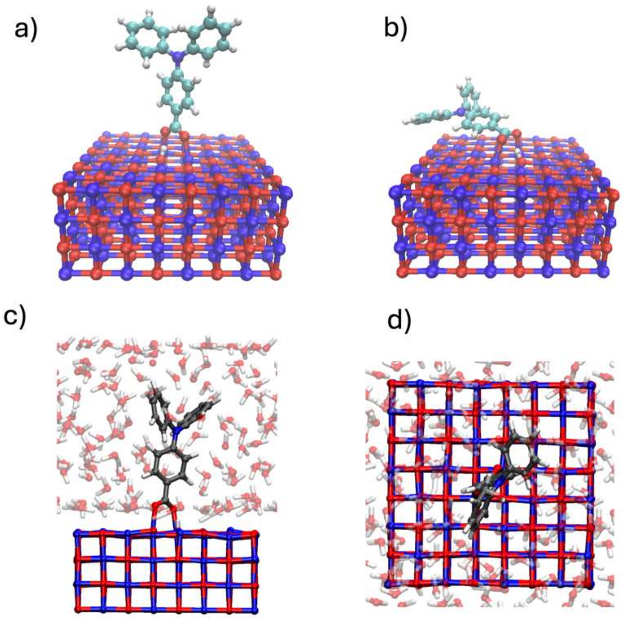

Figure S2. *a) and b) present visualizations of the 4DPABA dye in perpendicular orientation towards to surface and in when it lies on the surface, respectively. Top (c) and side (d) view of snapshots from the DFT-based molecular dynamics simulations of 4DPABA on NiO.*

SI.7 DFT optimization and TDDFT excitations spectrum of the P1 dye.

The P1 geometry was optimized at the DFT level using the AMS2021 program.[23] The optimizations employed the hybrid B3LYP exchange-correlations functional in an all-electron, triple-ζ (TZP) Slater-type basis. Van der Waals forces were corrected by the third generation of Grimme's dispersion corrections including Becke and Johnsen damping functions (D3BJ),[3–5,24] and relativistic effects were included by means of the Zeroth Order Regular Approximation (ZORA).[25] The COnductor like Screening MOdel (COSMO) was employed to take into account the electronic polarization effects of the water environment.[26–28] The numerical integration was improved over the default setting to "good", and the self-consistent field convergence was tightened to 10^{-8} H. A frequency calculation on the optimized P1 geometry did not reveal any imaginary eigenmodes, thereby verifying that the obtained structure represents a local minimum on the potential energy surface. The excitation spectrum was calculated by a Time-Dependent DFT (TDDFT) calculation within the Tamm-Dancoff formalism, employing the same settings as described above.[29]

The lowest 5 electronic excitations calculated by TDDFT are provided in Table S3. According to this calculation at the B3LYP level, the first bright (oscillator strength of 1.51 a.u.) excitation is largely (98.7%) described by the HOMO-LUMO transition.

Table S3. Unrestricted TDDFT (B3LYP-D3BJ/TZP) excitation spectrum of the P1 dye in implicit water solvation (COSMO). Also the first spin-forbidden singlet-triplet excitations are included.

	Spin	Excitation energy (eV)	Oscillator strength (a.u.)	Major MO contributions
1	singlet-triplet	1.73	0.05	α:156 →157 (53%), β:156 →157 (26%)
2	singlet-triplet	1.80	0.00	α:156 →158 (48%), β:156 →157 (22%)
3	singlet-singlet	2.15	1.51	β:156 →157 (64%) α:156 →157 (35%)
4	singlet-singlet	2.35	0.20	β:156 →158 (67%) α:156 →158 (29%)
5	singlet-triplet	2.42	0.01	α:155 →157 (30%), α:156 →158 (19%), β:155 →157 (17%), α:154 →158 (12%)

SI.8 Optimization of the Extended-Hückel parameters.

The simulations of electron and hole wave packets described in this work were performed within the framework of Extend-Hückel (EH) theory. A more elaborate overview of this method is provided for instance in ref.[30] The EH calculations on the NiO-P1 interface using the default EH parameters[31] did not yield a driving force for the hole transfer from the excited P1* to the NiO surface. A more realistic alignment of the P1 frontier orbitals with respect to the NiO valence band (VB), as well as a widening of the P1 HOMO-LUMO gap and a narrowing of the NiO band gap was achieved by fine-tuning the Coulomb integral parameters with a genetic algorithm. The optimization of the NiO parameters was performed on a bulk-phase, cubic NiO structure, containing 32 nickel and 32 oxygen atoms in a perfect rock-salt formation. Periodic boundary conditions were employed in the [100], [010] and [001] directions. The parameters for the P1 dye were optimized by performing the EH calculations on a DFT-optimized structure of the free P1 dye including the proton on the

carboxylate group, that would later dissociate upon anchoring to the NiO. During the optimization procedure, a distinction was made between different carbon and nitrogen atoms that were found in different chemical environments. A schematic representation of the different atomic species is provided in Figure S3a. In each optimization run, the genetic algorithm spawned a population of 50 sets of parameters of which the 2 best performing sets were propagated to the next generation. After 10 generations, the best performing set of parameters was assessed by hand to see whether the relevant orbitals still preserved the correct symmetry and spatial distribution. The target values for the optimization procedure are given in Table S4, while the formulation of the cost function is described in an earlier work.[30] Figure S3b provides a visual comparison between the frontier P1 orbitals calculated with DFT and with the final EH parameters. The obtained EH parameters for all chemical elements, as described in Figure S3a, are provided in the SI.11 appendix.

Table S4. The NiO VBM (valence band maximum), NiO CBM (conduction band minimum) and P1 energy level alignment with the original and optimized EH parameters.

	P1 HOMO (eV)	P1 LUMO (eV)	NiO VBM (eV)	NiO CBM (eV)
Original parameters	-11.2	-9.8	-12.3	-5.6
Optimized parameters	-11.4	-9.2	-10.4	-6.4
Optimization targets	-11.4	-9.2	-10.5	-6.5

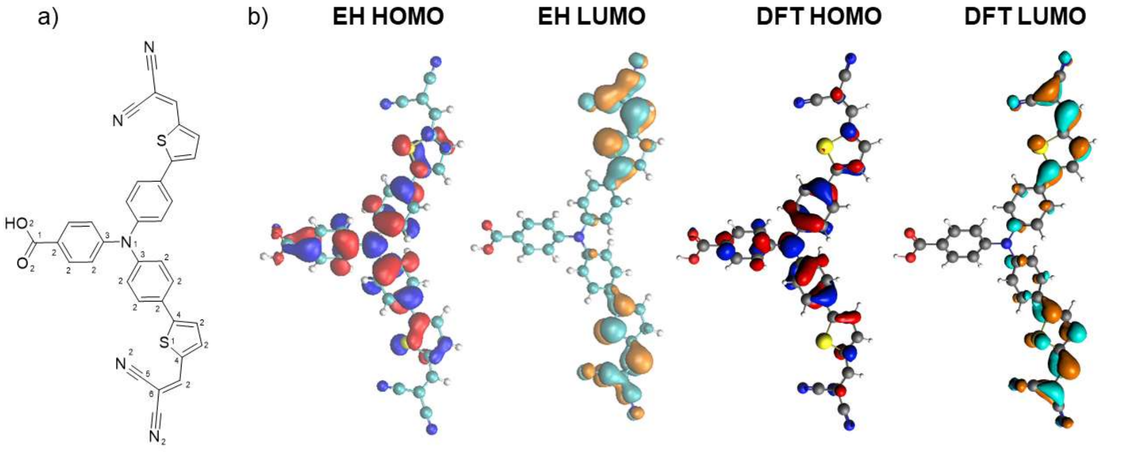

Figure S3. *a) provides the distinct atomic species in the EH optimization procedure. A visual comparison between the P1 HOMO and LUMO orbitals calculated at the DFT level and at the EH level is provided in b). The B3LYP functional was used for the DFT calculations*. Isosurfaces of the HOMO and LUMO orbitals were plotted at a density value of 0.03.

SI.9 Electron dynamics simulations on static structures and dynamics nuclear trajectories.

Figure S4 presents the electron survival probability on the excited P1* calculated from the quantum wave packet simulations on dynamic nuclear trajectories (green) and static nuclear structures (red). The three different panels correspond to the three initial conditions sampled from the performed classical MD simulations at *t*=0 fs, *t*=1000 fs and *t*=2000 fs. The electron wave packet appears to remain on the dye for the duration of the three quantum dynamics simulations on the dynamic trajectories. In contrast, the simulations on static structures show a gradual electron transfer to the NiO after the initial ~200 fs of the simulation. This suggests that after this initial period, a recombination channel becomes available which allows for the coherent charge transfer from the P1 LUMO to the NiO valence band. Interestingly, the nuclear motion appears to quench this coherent charge recombination channel.

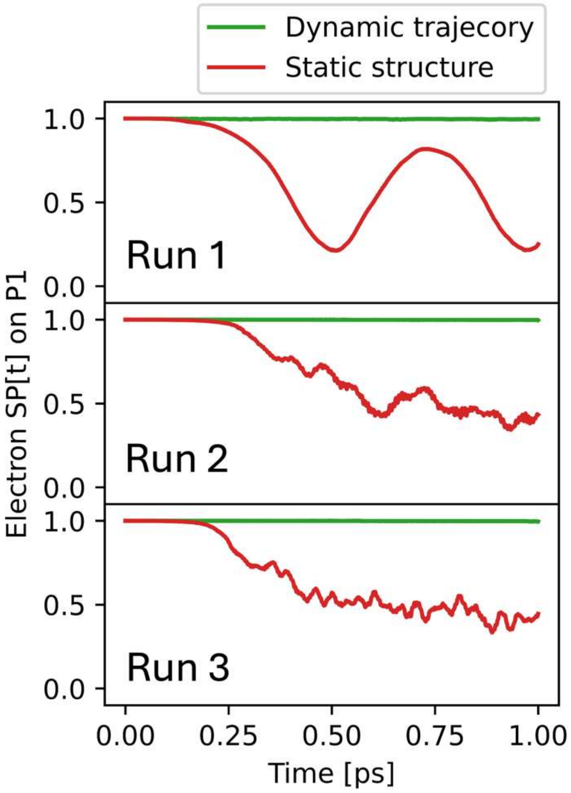

***Figure S4**: Time-dependent electron survival probability on the excited P1* from quantum wave packet simulations performed on static nuclear structures (red) and dynamic nuclear trajectories (green). The three panels correspond to three different simulations initiated at nuclear initial positions sampled from classical molecular dynamics simulations.*

SI.10 Ordering of HOMO and HOMO-1 states for different conformations sampled from MD.

Before performing the photoinduced hole injection quantum dynamics simulations, the relative alignment of the HOMO and HOMO-1 orbitals was investigated for a series of 30 different structures sampled from the MD simulations. For each of these structures, we performed two quantum dynamics simulations with the hole initialized in the HOMO or in the HOMO-1 orbital of the system. Subsequently, the hole density was visualized after the first 0.1 fs timestep. The orbital energies are plotted in Figure S5a, while two representative hole density visualizations are provided in Figure S5b. It was observed that for several conformational structures the HOMO and HOMO-1 orbitals become nearly degenerate and that for four structures their relative alignment is reversed.

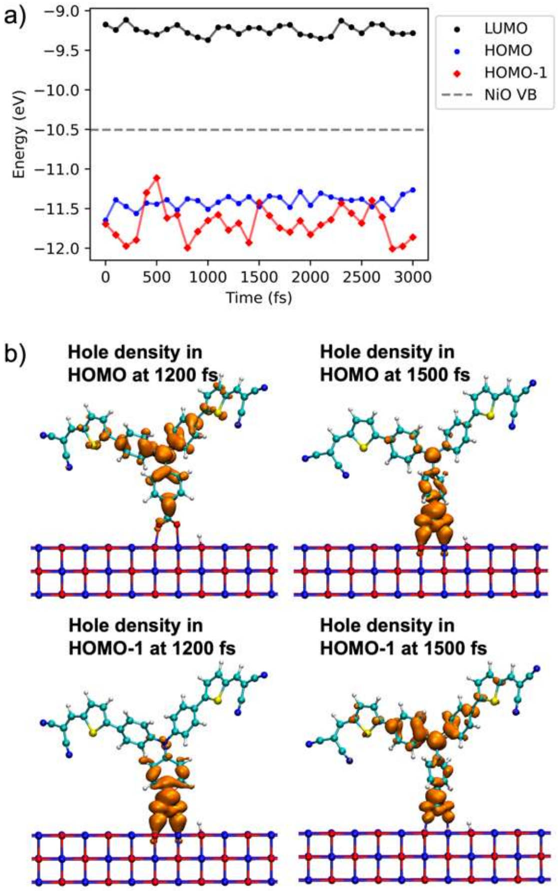

Figure S5. *a) The energies of the LUMO, HOMO and HOMO-1 states in the 3 ps time interval. b) Visualizations of the hole wave packet density initiated in the HOMO and HOMO-1 states at 1200 fs and 1500 fs. At this stage, the order of the energy levels is reversed, and the two states are mixed.*

SI.11 Extended Hückel-based DOS plot for P1 lying flat on surface.

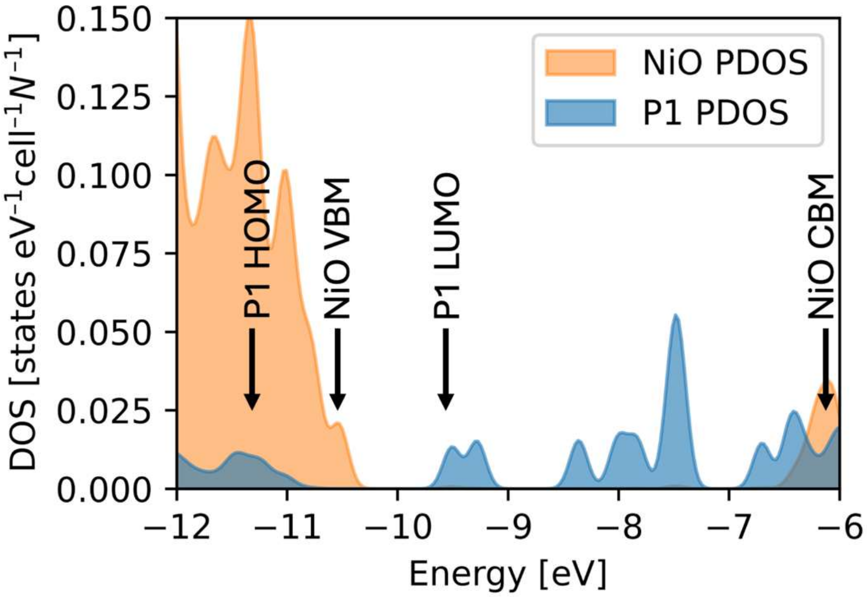

Figure S6. *Plot of the electronic density of states (DOS) of the optimized P1 dye lying flat on the NiO (100) slab (Species 2, Figure 4b).*

SI.12 Appendix: The optimized EH parameters.

Symbol	MMsymbol	NoAt	Nvalen	Nzeta	n	spdf	IP	zeta1	zeta2	coef1	coef2	k_WH
O	O1	8	6	1	2	s	-31.97708	2.419349	0.000000	1.000000	0.000000	2.388173
O	O1	8	6	1	2	p	-12.44601	2.188920	0.000000	1.000000	0.000000	1.708553
Ni	Ni1	28	10	1	4	s	-8.35691	2.423005	0.000000	1.000000	0.000000	1.726895
Ni	Ni1	28	10	1	4	p	-5.04010	1.550887	0.000000	1.000000	0.000000	3.040000
Ni	Ni1	28	10	2	3	d	-12.95211	5.881614	2.300000	0.568300	0.629200	2.007307
C	C1	6	4	1	2	s	-21.83998	1.502273	0.000000	1.000000	0.000000	1.652710
C	C1	6	4	1	2	p	-11.11776	1.578776	0.000000	1.000000	0.000000	1.271570
C	C2	6	4	1	2	s	-21.64263	1.392992	0.000000	1.000000	0.000000	1.586950
C	C2	6	4	1	2	p	-11.30164	1.592068	0.000000	1.000000	0.000000	2.189580
C	C3	6	4	1	2	s	-21.30227	1.602251	0.000000	1.000000	0.000000	1.399880
C	C3	6	4	1	2	p	-12.56206	1.907199	0.000000	1.000000	0.000000	1.909580
C	C4	6	4	1	2	s	-21.26181	1.652660	0.000000	1.000000	0.000000	1.905050
C	C4	6	4	1	2	p	-11.55636	1.727973	0.000000	1.000000	0.000000	1.932090
C	C5	6	4	1	2	s	-21.79423	1.641574	0.000000	1.000000	0.000000	1.521610
C	C5	6	4	1	2	p	-10.69162	1.507971	0.000000	1.000000	0.000000	2.166900
C	C6	6	4	1	2	s	-21.66886	1.537034	0.000000	1.000000	0.000000	1.718590
C	C6	6	4	1	2	p	-11.56014	1.599292	0.000000	1.000000	0.000000	1.678440
S	S1	16	6	1	3	s	-20.14530	2.069794	0.000000	1.000000	0.000000	2.032820
S	S1	16	6	1	3	p	-11.89877	1.899772	0.000000	1.000000	0.000000	3.837720
N	N1	7	5	1	2	s	-25.46722	2.010008	0.000000	1.000000	0.000000	1.282550
N	N1	7	5	1	2	p	-12.73100	2.281318	0.000000	1.000000	0.000000	1.988070
N	N2	7	5	1	2	s	-26.01483	1.859309	0.000000	1.000000	0.000000	1.817200
N	N2	7	5	1	2	p	-13.46475	2.227766	0.000000	1.000000	0.000000	1.427180
O	O2	8	6	1	2	s	-32.51769	1.823570	0.000000	1.000000	0.000000	1.869520
O	O2	8	6	1	2	p	-14.66009	1.962404	0.000000	1.000000	0.000000	2.132110

References

(1) Kühne, T. D.; Iannuzzi, M.; Del Ben, M.; Rybkin, V. V.; Seewald, P.; Stein, F.; Laino, T.; Khaliullin, R. Z.; Schütt, O.; Schiffmann,; et al. CP2K: An Electronic Structure and Molecular Dynamics Software Package - Quickstep: Efficient and Accurate Electronic Structure Calculations. *J. Chem. Phys.* **2020**, *152* (19), 194103. https://doi.org/10.1063/5.0007045.

(2) Perdew, J. P.; Burke, K.; Ernzerhof, M. Generalized Gradient Approximation Made Simple [Phys. Rev. Lett. 77, 3865 (1996)]. *Phys. Rev. Lett.* **1997**, *78* (7), 1396–1396. https://doi.org/10.1103/PhysRevLett.78.1396.

(3) Grimme, S. Accurate Description of van Der Waals Complexes by Density Functional Theory Including Empirical Corrections. *J. Comput. Chem.* **2004**, *25* (12), 1463–1473. https://doi.org/10.1002/jcc.20078.

(4) Grimme, S. Density Functional Theory with London Dispersion Corrections. Wiley *Interdiscip. Rev. Comput. Mol. Sci.* **2011**, *1* (2), 211–228. https://doi.org/10.1002/wcms.30.

(5) Grimme, S. Supramolecular Binding Thermodynamics by Dispersion-Corrected Density Functional Theory. *Chem. - Eur. J.* **2012**, *18* (32), 9955–9964. https://doi.org/10.1002/chem.201200497.

(6) VandeVondele, J.; Krack, M.; Mohamed, F.; Parrinello, M.; Chassaing, T.; Hutter, J. Quickstep: Fast and Accurate Density Functional Calculations Using a Mixed Gaussian and Plane Waves Approach. *Comput. Phys. Commun.* **2005**, *167* (2), 103–128. https://doi.org/10.1016/j.cpc.2004.12.014.

(7) Goedecker, S.; Teter, M.; Hutter, J. Separable Dual-Space Gaussian Pseudopotentials. *Phys. Rev. B* **1996**, *54* (3), 1703–1710. https://doi.org/10.1103/PhysRevB.54.1703.

(8) Hartwigsen, C.; Goedecker, S.; Hutter, J. Relativistic Separable Dual-Space Gaussian Pseudopotentials from H to Rn. *Phys. Rev. B* **1998**, *58* (7), 3641–3662. https://doi.org/10.1103/PhysRevB.58.3641.

(9) Krack, M. Pseudopotentials for H to Kr Optimized for Gradient-Corrected Exchange-Correlation Functionals. *Theor. Chem. Acc.* **2005**, *114* (1–3), 145–152. https://doi.org/10.1007/s00214-005-0655-y.

(10) Cairns, R. W.; Ott, E. X-Ray Studies of the System Nickel—Oxygen—Water. I. Nickelous Oxide and Hydroxide [1]. *J. Am. Chem. Soc.* **1933**, *55* (2), 527–533. https://doi.org/10.1021/ja01329a013.

(11) Cheetham, A. K.; Hope, D. A. O. Magnetic Ordering and Exchange Effects in the Antiferromagnetic Solid Solutions Mn x Ni 1 − x O. *Phys. Rev. B* **1983**, *27* (11), 6964–6967. https://doi.org/10.1103/PhysRevB.27.6964.

(12) Walls, B.; Mazilkin, A. A.; Mukhamedov, B. O.; Ionov, A.; Smirnova, I. A.; Ponomareva, A. V.; Fleischer, K.; Kozlovskaya, N. A.; Shulyatev, D. A.; Abrikosov, I. A.; Shvets, I. V.; Bozhko, S. I. Nanodomain Structure of Single Crystalline Nickel Oxide. *Sci. Rep.* **2021**, *11* (1), 3496. https://doi.org/10.1038/s41598-021-82070-1.

(13) Piccinin, S.; Rocca, D.; Pastore, M. Role of Solvent in the Energy Level Alignment of Dye-Sensitized NiO Interfaces. *J. Phys. Chem. C* **2017**, *121* (40), 22286–22294. https://doi.org/10.1021/acs.jpcc.7b08463.

(14) Segalina, A.; Lebègue, S.; Rocca, D.; Piccinin, S.; Pastore, M. Structure and Energetics of Dye-Sensitized NiO Interfaces in Water from Ab Initio MD and Large-Scale GW Calculations. *J. Chem. Theory Comput.* **2021**, *17* (8), 5225–5238. https://doi.org/10.1021/acs.jctc.1c00354.

(15) Massin, J.; Bräutigam, M.; Bold, S.; Wächtler, M.; Pavone, M.; Muñoz-García, A. B.; Dietzek, B.; Artero, V.; Chavarot-Kerlidou, M. Investigating Light-Driven Hole

Injection and Hydrogen Evolution Catalysis at Dye-Sensitized NiO Photocathodes: A Combined Experimental-Theoretical Study. *J. Phys. Chem. C* **2019**, *123* (28), 17176–17184. https://doi.org/10.1021/acs.jpcc.9b04715.

(16) Muñoz-García, A. B.; Pavone, M. Structure and Energy Level Alignment at the Dye–Electrode Interface in p-Type DSSCs: New Hints on the Role of Anchoring Modes from Ab Initio Calculations. *Phys. Chem. Chem. Phys.* **2015**, *17* (18), 12238–12246. https://doi.org/10.1039/C5CP01020A.

(17) Zhao, W.; Doyle, A. D.; Morgan, S. E.; Bajdich, M.; Nørskov, J. K.; Campbell, C. T. Formic Acid Dissociative Adsorption on NiO(111): Energetics and Structure of Adsorbed Formate. *J. Phys. Chem. C* **2017**, *121* (50), 28001–28006. https://doi.org/10.1021/acs.jpcc.7b09405.

(18) Bussi, G.; Donadio, D.; Parrinello, M. Canonical Sampling through Velocity Rescaling. *J. Chem. Phys.* **2007**, *126* (1), 014101. https://doi.org/10.1063/1.2408420.

(19) Torres, A.; Oliboni, R. S.; Rego, L. G. C. Vibronic and Coherent Effects on Interfacial Electron Transfer Dynamics. *J. Phys. Chem. Lett.* **2015**.

(20) Da Silva, R.; Hoff, D. A.; Rego, L. G. C. Coupled Quantum-Classical Method for Long Range Charge Transfer: Relevance of the Nuclear Motion to the Quantum Electron Dynamics. *J. Phys.: Condens. Matter* **2015**, *27* (13), 134206. https://doi.org/10.1088/0953-8984/27/13/134206.

(21) Rego, L. G. C.; Batista, V. S. Quantum Dynamics Simulations of Interfacial Electron Transfer in Sensitized TiO2 Semiconductors. *J. Am. Chem. Soc.* **2003**, *125* (26), 7989–7997. https://doi.org/10.1021/ja0346330.

(22) Martínez, L.; Andrade, R.; Birgin, E. G.; Martínez, J. M. P ACKMOL : A Package for Building Initial Configurations for Molecular Dynamics Simulations. *J. Comput. Chem.* **2009**, *30* (13), 2157–2164. https://doi.org/10.1002/jcc.21224.

(23) R. Rüger, M. Franchini, T. Trnka, A. Yakovlev, E. van Lenthe, P. Philipsen, T. van Vuren, B. Klumpers, T. Soini. AMS 2021.101, 2021. http://www.scm.com.

(24) Grimme, S.; Ehrlich, S.; Goerigk, L. Effect of the Damping Function in Dispersion Corrected Density Functional Theory. *J. Comput. Chem.* **2011**, *32* (7), 1456–1465. https://doi.org/10.1002/jcc.21759.

(25) Van Lenthe, E. Geometry Optimizations in the Zero Order Regular Approximation for Relativistic Effects. *J. Chem. Phys.* **1999**, *110* (18), 8943–8953. https://doi.org/10.1063/1.478813.

(26) Klamt, A. Conductor-like Screening Model for Real Solvents: A New Approach to the Quantitative Calculation of Solvation Phenomena. *J. Phys. Chem.* **1995**, *99* (7), 2224–2235. https://doi.org/10.1021/j100007a062.

(27) Klamt, A.; Jonas, V. Treatment of the Outlying Charge in Continuum Solvation Models. *J. Chem. Phys.* **1996**, *105* (22), 9972–9981. https://doi.org/10.1063/1.472829.

(28) Pye, C. C.; Ziegler, T.; Van Lenthe, E.; Louwen, J. N. An Implementation of the Conductor-like Screening Model of Solvation within the Amsterdam Density Functional Package — Part II. COSMO for Real Solvents1. *Can. J. Chem* **2009**, *87*, 790–797. https://doi.org/doi:10.1139/V09-008.

(29) Hirata, S.; Head-Gordon, M. Time-Dependent Density Functional Theory within the Tamm-Dancoff Approximation. *Chem. Phys. Lett.* **1999**, *314* (3–4), 291–299. https://doi.org/10.1016/S0009-2614(99)01149-5.

(30) Monti, A.; Negre, C. F. A.; Batista, V. S.; Rego, L. G. C.; De Groot, H. J. M.; Buda, F. Crucial Role of Nuclear Dynamics for Electron Injection in a Dye-Semiconductor Complex. *J. Phys. Chem. Lett.* **2015**, *6* (12), 2393–2398. https://doi.org/10.1021/acs.jpclett.5b00876.

(31) Santiago Alvarez. Table of Parameters for Extended Hückel Calculations', Universitat de Barcelona: Barcelona, 1993.
